# Supplementary material for: Epigenetic modulation of inflammation and synaptic plasticity promotes resilience against stress in mice
Source: Nat Commun. 2018 Feb 2;9:477. doi: 10.1038/s41467-017-02794-5 (PMC5797143; doi:10.1038/s41467-017-02794-5)
Supplement: Supplementary file 1 — Supplementary Information [file 41467_2017_2794_MOESM1_ESM.pdf]

Supplementary Information

Epigenetic modulation of inflammation and synaptic plasticity promotes resilience against stress in mice

Wang *et. al.*

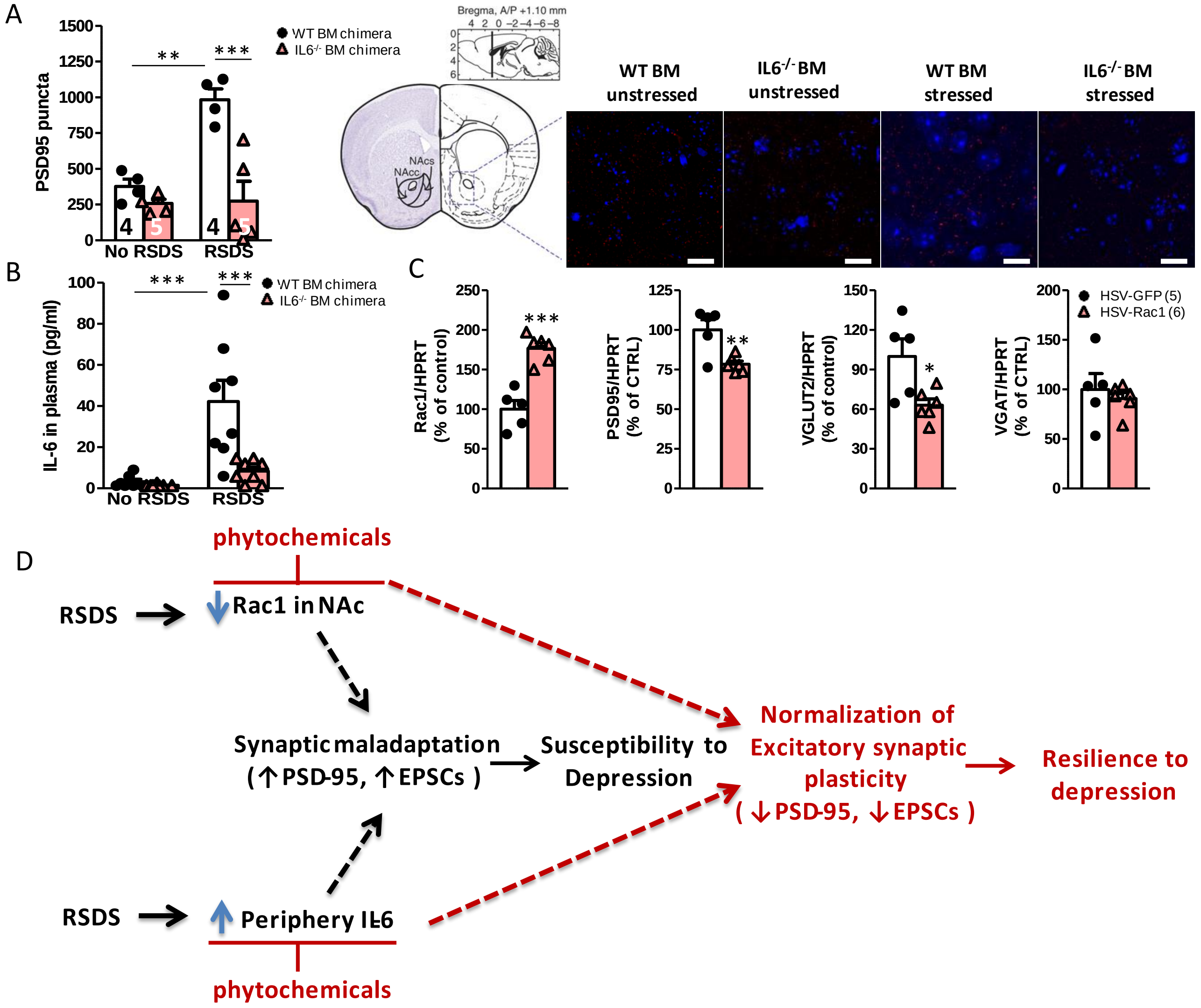

Supplementary Figure 1. The role of IL-6 and Rac1 in modulating synaptic plasticity. (**A**) Quantification of marker of excitatory synapses PSD-95 puncta in NAc from WT and IL-6$^{-/-}$ BM chimeric mice with or without RSDS (one-way ANOVA, $F_{3,17}$ =13.94, P=0.0002). Inset: representative schematic of mouse NAc region used for the immunohistological quantification and representative images showing increased frequency of PSD-95 puncta in NAc of stressed WT BM chimeras compared to unstressed WT chimeras and stressed or unstressed IL-6$^{-/-}$ BM chimeric mice; scale bar = 10µm. (**B**) Measurements of IL-6 in the plasma following RSDS (one-way ANOVA, $F_{3,30}$ =12.52, P<0.0001, n=8,8,7,8 mice (**C**) Modulation of synaptic markers on excitatory neurons by Rac1 in MSN-enriched primary cultures. Gene expression of glutamatergic PSD-95, GLUT2 and GABAergic VGAT in MSNs 48 hours following HSV-GFP or HSV-Rac1 infection (unpaired two tailed *t*-test). (D) Working hypothesis: RSDS–mediated down regulation of Rac1 in the NAc and upregulation of peripheral IL-6 lead to synaptic maladaptation in the NAc and susceptible phenotype of depression (Black). Select phytochemicals can block RSDS-mediated changes of Rac1 and IL-6, thus normalizing synaptic plasticity and promoting resilience to stress-induced depression (Red). All graphs represent mean ± s.e.m., *P<0.05, **P<0.01, ***P<0.001

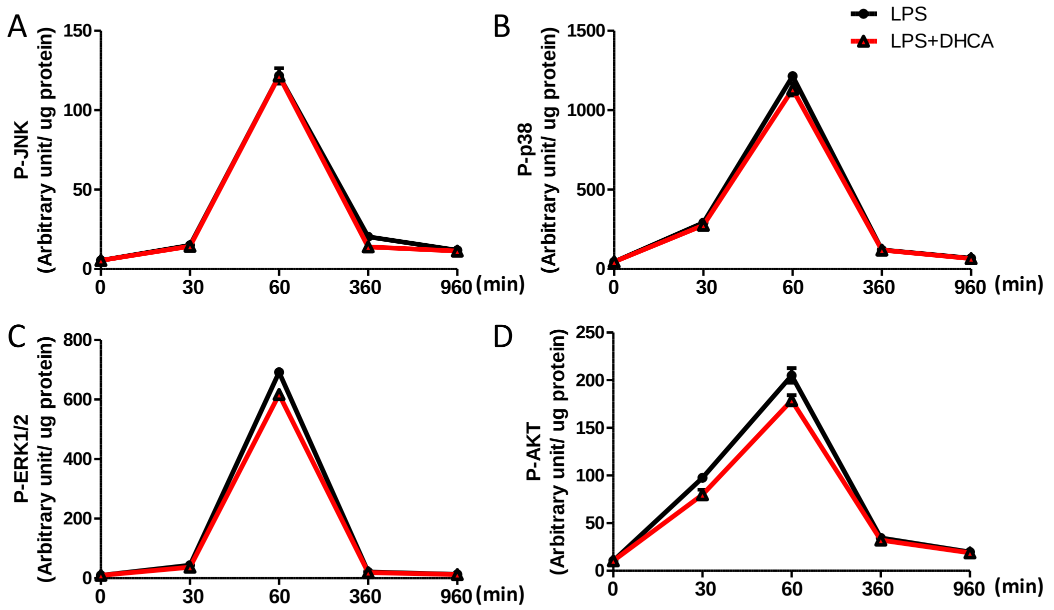

Supplementary Figure 2. The effect of DHCA on signal transduction pathways in PBMCs following LPS stimulation. The expression of (**A**) p-JNK (pT183/pY185), (**B**) p-p38 (pT180/pY182), (**C**) p-ERK1/2 (pT185/pY187) and (**D**) p-AKT (pS473) in PBMCs following 0, 30 minutes, 60 minutes, 360 minutes and 960 minutes of LPS stimulation in the presence or absence of overnight treatment with DHCA (Two-way ANOVA $P=0.202$ $F_{4,30}=2.05$ for p-JNK; $P=0.067$ $F_{4,30}=4.98$ for p-p38; $P=0.010$, $F_{4,30}=14.41$ for p-ERK1/2 and $P=0.013$, $F_{4,30}=12.05$ for p-AKT, n=4 per culture per condition)

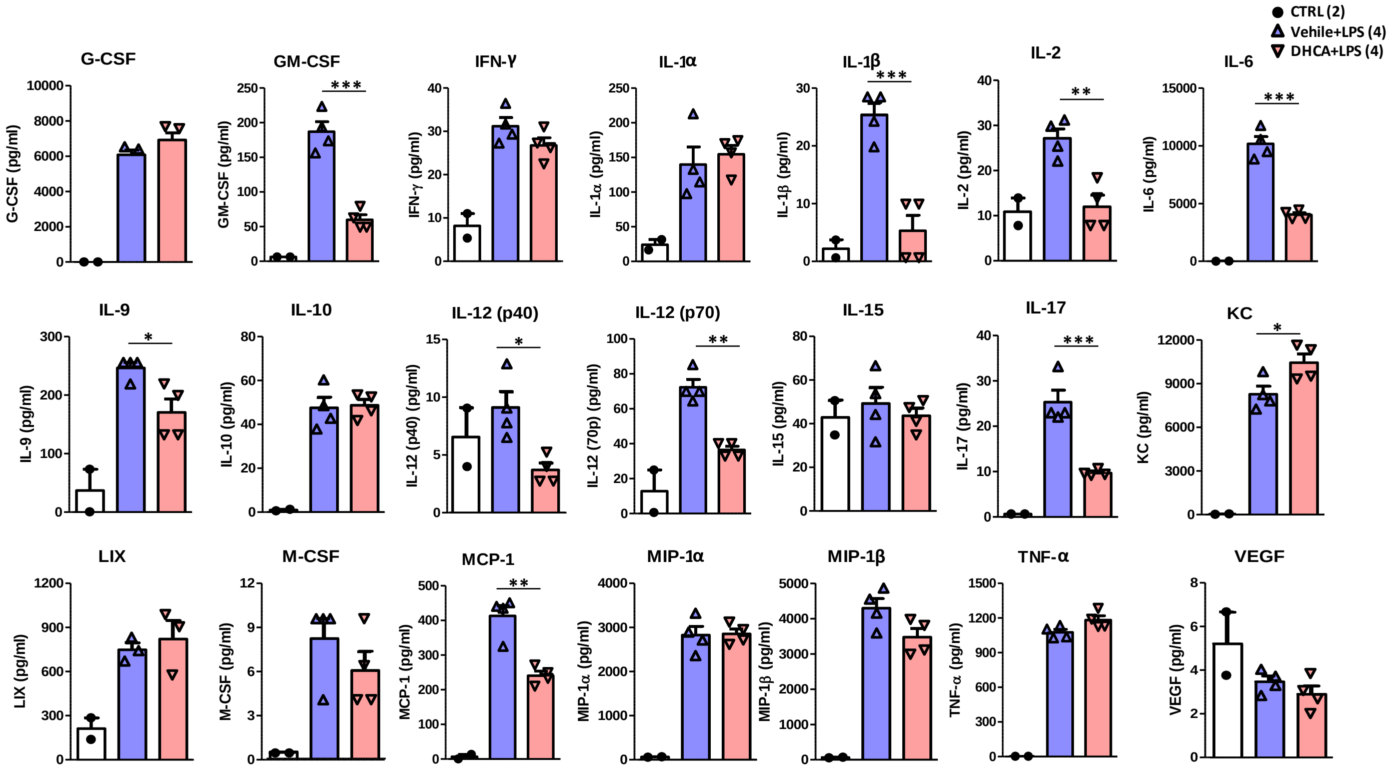

Supplementary Figure 3. Secretion of cytokines from PBMCs following LPS stimulation. Mouse PBMCs treated with vehicle or DHCA were stimulated with LPS for 16 hours and cytokines were measured using Mouse Cytokine/chemokine Magnetic Bead Panel Milliplex MAP kit. One-way ANOVA, **P*<0.05, ***P*<0.01, ****P*<0.001. All graphs represent mean ± s.e.m..

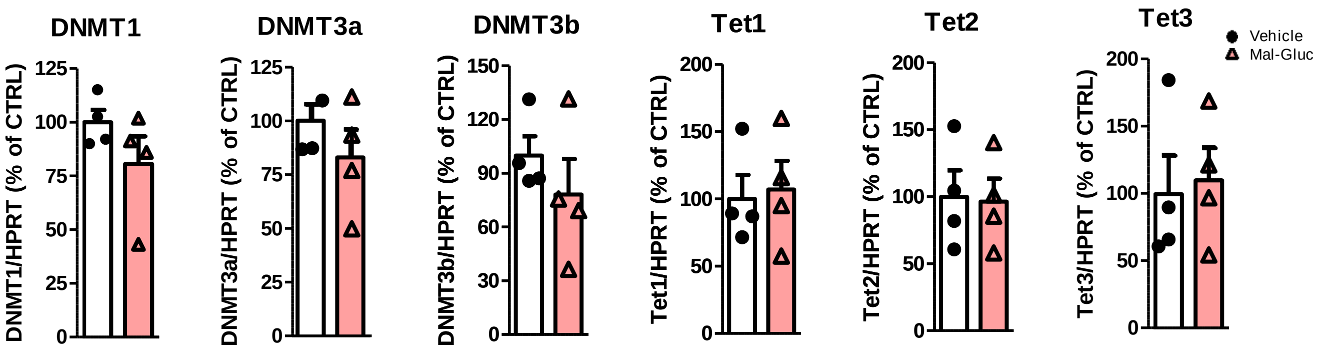

Supplementary Figure 4. Mal-gluc treatment of MSN-enriched primary neurons has no effect on the expression of genes important for DNA methylation. The expression of DNMT1, DNMT31, DNMT3b, Tet1, Tet2 and Tet3 in MSN-enriched primary neurons following 48 hours treatment with Mal-gluc by real-time PCR (two-tailed unpaired *t*-test, t_6 =1.372, P=0.219 for DNMT1; t_6 =1.135, P=0.300 for DNMT3a; t_6 =0.969, P=0.370 for DNMT3b; t_6 =0.256, P=0.806 for Tet1; t_6 =0.139, P=0.894 for Tet2; t_6 =0.274, P=0.793 for Tet3, n=4 per culture). All graphs represent mean ± s.e.m..

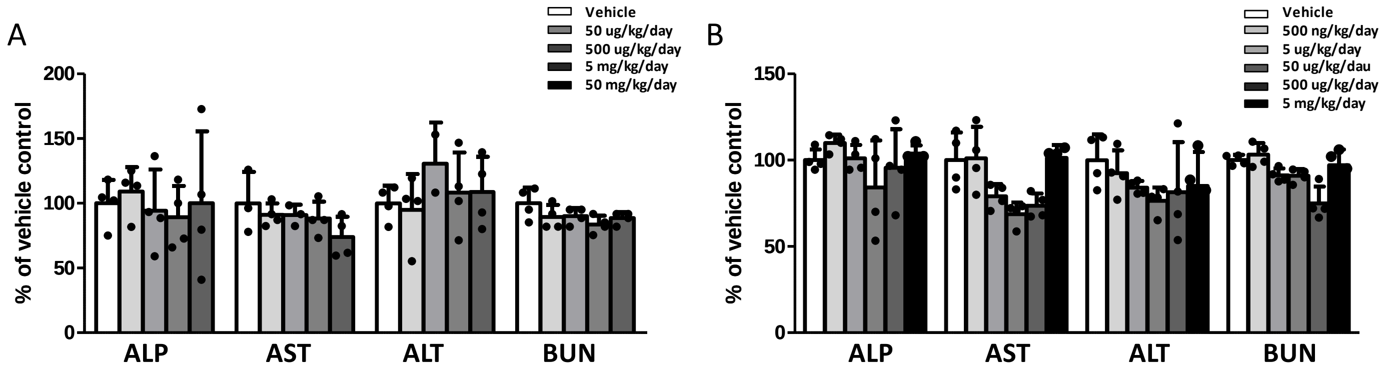

Supplementary Figure 5. In vivo toxicology indexes in C57BL/6 mice follwoing two-week treatment with various doses of DHCA or Mal-gluc (**A**) Blood level of ALP, AST, ALT and BUN following oral administration of DHCA treatment (one-way ANOVA, $F_{4,19}$ =0.204, P=0.932 for ALP; $F_{4,19}$ =0.981, P=0.447 for AST; $F_{4,17}$ =1.491, P=0.362 for ALT; $F_{4,19}$ =2.095, P=0.132 for BUN; n=4 animals per dose). (**B**) Blood level of ALP, AST, ALT and BUN following oral administration of Mal-gluc treatment (one-way ANOVA, $F_{5,23}$ =0.63, P=0.673 for ALP; $F_{5,23}$= 1.828, P=0.158 for AST; $F_{5,23}$= 0.258, P=0.930 for ALT; $F_{5,23}$= 2.398, P=0.078 for BUN, n=4 animals per dose). All graphs represent mean ± s.e.m..

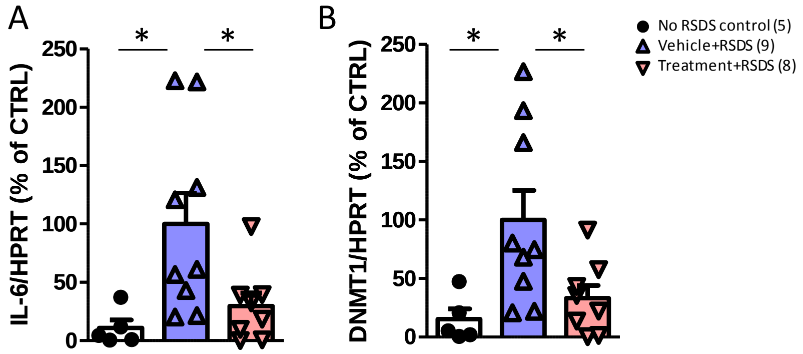

Supplementary Figure 6. Expression of *IL-6* and *DNMT1* in PBMCs isolated from non-stressed control mice and stressed mice with or without DHCA/Mal-gluc treatment (**A**) Measurements of *IL-6* mRNA level by real time PCR (one-way ANOVA, $F_{2,21}$ =5.363, P=0.0142). (**A**) Measurements of *DNMT1* mRNA level by real time PCR (one-way ANOVA, $F_{2,21}$ =5.207, P=0.0157). *P<0.05. All graphs represent mean ± s.e.m..

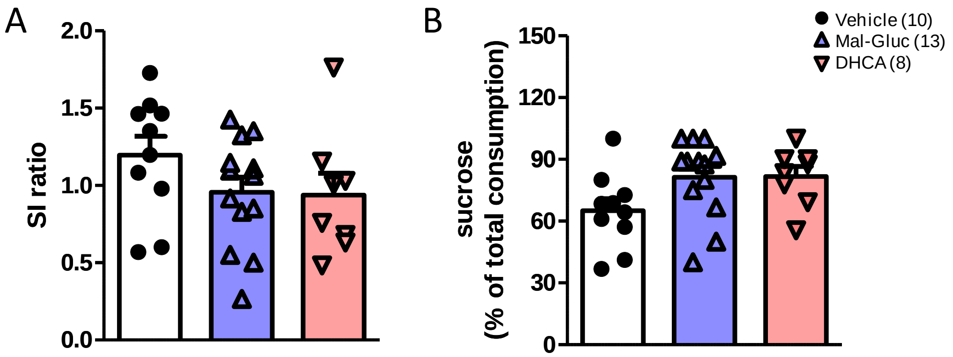

Supplementary Figure 7. Single target compound treatment does not attenuate depression-like phenotypes following RSDS. (**A**) Social avoidance behavioral test in mice treated with either Mal-gluc or DHCA two weeks prior to RSDS and throughout the RSDS (one-way ANOVA, $F_{2,30}$ =1.451, P=0.251). (**B**) Sucrose preference test following SI test (One-way ANOVA, $F_{2,30}$ =2.961, P=0.068). All graphs represent mean ± s.e.m..

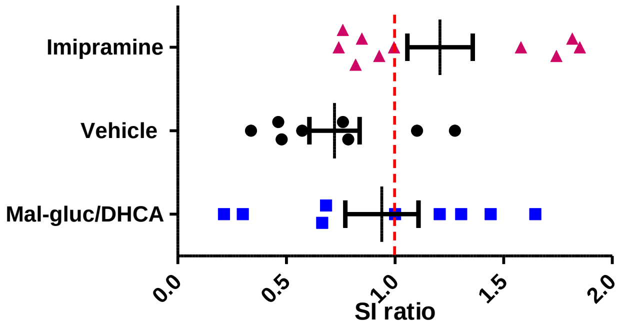

Supplementary Figure 8. Imipramine and Mal-gluc/DHCA have similar therapeutic efficacy in treating animals with depression-like social avoidance behavior. Re-testing of social avoidance behavior in susceptible mice following treatment with imipramine, Mal-gluc/DHCA or vehicle (one-way ANOVA, $F_{2,26}$ =2.652, P=0.0911, n=10, 8, 9 mice). Graphs represents mean ± s.e.m..

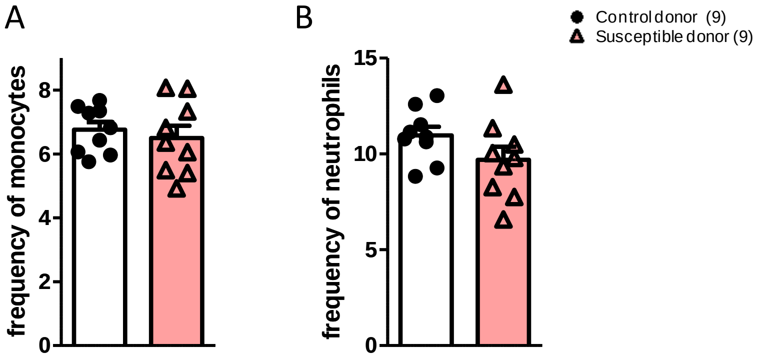

Supplementary Figure 9. The susceptibility of the donor mice has no significant effect on the regeneration of monocytes or nerutophils prior to sub-threshhold defeat. (A) The frequency of total monocytes and (B) The frequency of total neutorphils in the blood of chimeras either from control donor or from susceptible donor without subthreshhold defeat (two-tailed unpaired *t*-test, t_{16} =0.570, *P*=0.576 for monocytes and t_{16} =1.541, *P*=0.143 for neutrophils). All graphs represent mean ± s.e.m..

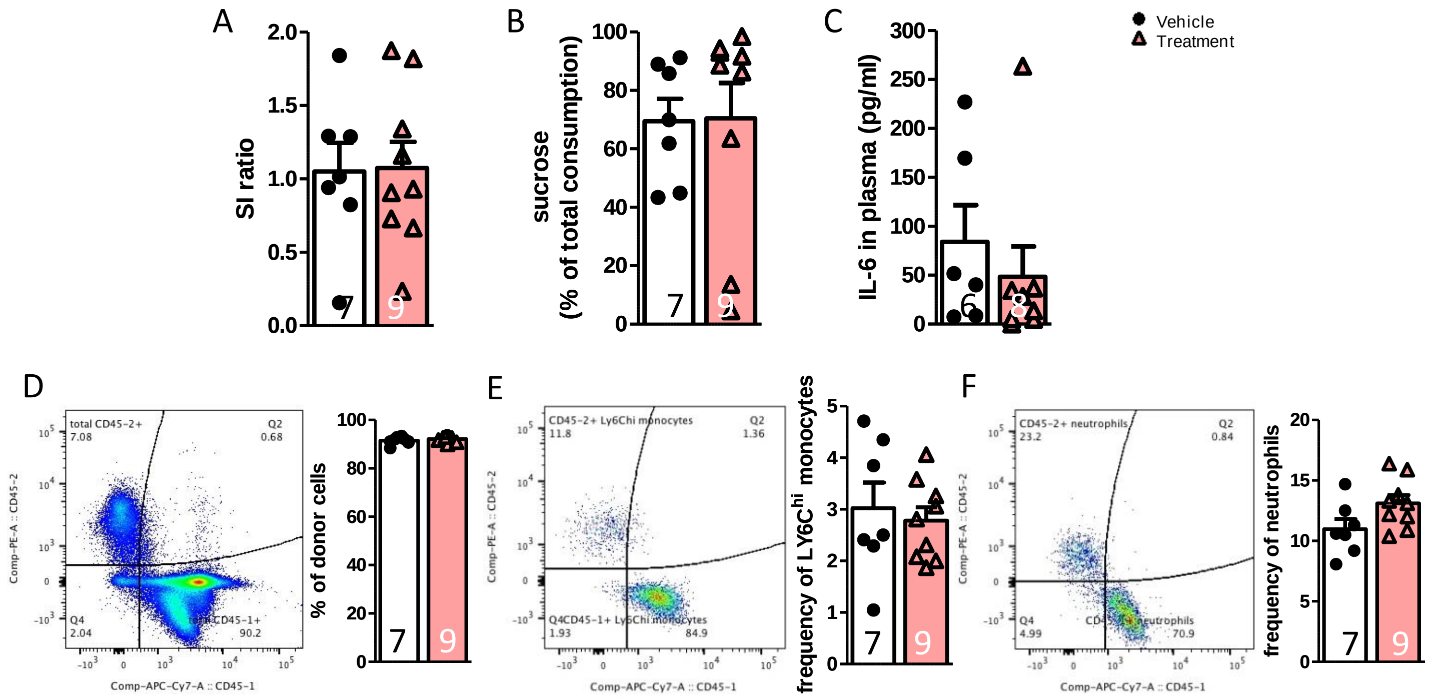

Supplementary Figure 10. DHCA/Mal-gluc treatment has no significant effect on behavioral and blood cell composition in chimeras with BM reconstructed from naïve mice. (**A**) Social avoidance test (two-tailed unpaired *t*-test, t_{14} =4.58, *P*=0.929). (**B**) Sucrose preference test (two-tailed unpaired *t*-test, t_{14} =0.0138, *P*=0.989). (**C**) Plasma level of IL-6 24 hours after the sub-threshold defeat (two-tailed unpaired *t*-test, t_{14} =0.736, *P*=0.476). (**D**) Representative image of flow cytometry gating for donor and recipient viable cells and percentage of cells derived from the donor (two-tailed unpaired *t*-test, t_{14} =1.661, *P*=0.115). (**E**) Representative image of flow cytometry gating for monocytes and frequency of monocytes of donor origin. Numbers represent percentages of live cells (two-tailed unpaired *t*-test, t_{14} =0.462, *P*=0.651). (**F**) Representative image of flow cytometry gating for neutrophils and frequency of neutrophils of donor origin. Numbers represent percentages of live cells (two-tailed unpaired *t*-test, t_{14} =2.014, *P*=0.064). All graphs represent mean ± s.e.m..

		Biologically Available Phenolic Metabolotes	Detection in Plasma	Detection in Brain	Compounds Screened
Polyphenol Metabolites	1	resveratrol	+	+	+
	2	resveratrol-glucuronide	+	+	+
	3	3'-OME-catechin-5-glucuronide	+	+	N/A
	4	3'-OME-epicatechin-5-glucuronide	+	+	+
	5	catechin-5-glucuronide	+	+	N/A
	6	delphinidin-glucuronide	+	+	N/A
	7	epicatechin-5-glucuronide	+	+	N/A
	8	quercetin-glucuroide	+	+	+
	9	OMe-quercetin-glucuronide	+	+	N/A
	10	OMe-resveratrol-glucuronide	+	+	N/A
	11	cyanidin-3-glucoside	+	+	+
	12	delphinidin-3-glucoside	+	+	+
	13	malvidin-3-glucoside	+	+	+
	14	peonidin-3-glucoside	+	+	N/A
Phenolic Acids	15	3-hydroxybenzoic acid	-	+	+
	16	3-(3´-hydroxyphenyl) propionic acid	+	+	+
	17	homovanilic acid	+	-	+
	18	3',4'-dihydrocaffeic acid	+	-	+
	19	5-(4´-hydroxyphenyl)valeric acid	+	-	+
	20	3-hydroxyphenylacetic acid	+	-	+
	21	ferulic acid-4'-O-sulfate	+	-	+

Supplementary Table 1. Biologically available BDPP phenolic metabolites. List of 14 polyphenol metabolites and 7 phenolic acids found accumulated in plasma and/or brain following oral administration of BDPP or BDPP components. Fourteen of the compounds were available, either through commercial sources or our own biosynthesis, for our in vitro investigations. + denotes phenolic metabolites screened in Figs. 2A, 2B, 2C and 2D; N/A denotes phenolic metabolites that are not available for our investigation.

Gene	Forward	Reverse
Rac1	GGTAGGTGATGGGAGTCAGC	CTGAAGTGCGACACCACTGT
PSD95	CGGGAGAAAATGGAGAAGGAC	GCATTGGCTGAGACATCAAG
VGLUT2	GCTCACCTCTACCCTCAATATG	CCACTTGCTCCATATCCCATG
VGAT	ACGACAAACCCAAGATCACG	AAGATGATGAGGAACAACCCC
HPRT	CCCCAAAATGGTTAAGGTTGC	AACAAAGTCTGGCCTGTATCC
DNMT1	CTCAGGGACCATATCTGCAAG	GGTGTACTGTAGCTTATGGGC
DNMT3a	GGAAAGATCATGTACGTCGGG	GCCAGTACCCTCATAAAGTCC
DNMT3b	GTACCCCATCAGTTGACTTGAG	TTGATCTTTCCCCACACGAG
TET1	GAGCCTGTTCCTCGATGTGG	CAAACCCACCTGAGGCTGTT
TET2	TGTTGTTGTCAGGGTGAGAATC	TCTTGCTTCTGGCAAACTTACA
TET3	CCGGATTGAGAAGGTCATCTAC	AAGATAACAATCACGGCGTTC
HDAC2	GGGACAGGCTTGGTTGTTTC	GAGCATCAGCAATGGCAAGT
HDAC3	TGTCTCAATGTGCCCTTACG	CCTAATCGATCACAGCCCAG
HDAC4	CAATCCCACAGTCTCCGTGT	CAGCACCCCACTAAGGTTCA
HDAC5	TTCAACTCCGTAGCCATCAC	GGATCGTTGTAGAATGCTTGC
HDAC7	GGTGGACCCCCCTTTCAGAAG	TGGGTAGCCAGGAGTCTGGA
HDAC9	GCGAGACACAGATGCTCAGAC	TGGGTTTTCCTTCCATTGCT

Supplementary Table 2. Mouse qPCR primer sets used in the study.
